# 219. Outcomes with Low- vs. High-bioavailability Oral Antibiotics in Treatment of Uncomplicated Gram-Negative Bacteremia

**DOI:** 10.1093/ofid/ofab466.421

**Published:** 2021-12-04

**Authors:** Ashley L Cubillos, Elisabeth Chandler, Ian P Murphy, Robert Castro

**Affiliations:** Lee Health, Fort Myers, Florida

## Abstract

**Background:**

High-bioavailability (HIGH-BIO) oral agents (i.e. trimethoprim-sulfamethoxazole, fluoroquinolones) are increasingly utilized for definitive treatment of uncomplicated gram-negative (UGN) bloodstream infections (BSI). Literature supports use of HIGH-BIO agents as step-down therapy, but few studies have assessed use of low-bioavailability (LOW-BIO) agents (i.e. beta-lactams). Increased recurrence of BSI has been associated with LOW-BIO agents; suboptimal dosing of beta-lactam agents may have impacted outcomes. Trials have not assessed whether high-dose beta-lactams (HD-BL) improve clinical outcomes over low-dose beta-lactams (LD-BL) for UGN BSI.

**Methods:**

This retrospective cohort study conducted between December 2016 and December 2020 included adults with UGN BSI administered oral step-down therapy for at least 1/3^rd^ the total antibiotic duration. The primary outcome was incidence of treatment failure of HIGH-BIO compared to LOW-BIO agents within 90 days of completing oral therapy. Treatment failure was a composite of all-cause mortality, recurrent BSI, reinfection of the primary site, or transition to IV antibiotics after initiating oral therapy. Secondary outcomes were incidence of treatment failure of HIGH-BIO compared to HD-BL agents, and of HD-BL compared to LD-BL agents.

**Results:**

Of 225 patients, 67 (29.8%) received a HIGH-BIO and 158 (70.2%) a LOW-BIO agent; of those in the LOW-BIO arm 126 (79.7%) received a HD-BL. The most common source of BSI was urinary (202 [89.8%]); transition to oral therapy occurred after a mean of 5 ± 2.39 days. No difference in treatment failure was observed (8 [11.9%] HIGH-BIO vs. 25 [15.8%] LOW-BIO, P = 0.45). A numerically higher number of patients in the LOW-BIO arm had recurrent BSI (4 [2.5%] LOW-BIO vs. 0 [0%] HIGH-BIO, P = 0.18). No difference in treatment failure was observed between HIGH-BIO and HD-BL agents (8 [11.9%] vs. 20 [15.9%], P = 0.46), or HD-BL and LD-BL agents (20 [15.9%] vs. 5 [15.6%], P = 0.97).

**Conclusion:**

No difference in treatment failure was observed between groups; further study is needed due to failure to reach statistical power. A numerical trend towards increased recurrence of BSI was observed with LOW-BIO agents. Beta-lactams may be reasonable for step-down therapy of UGN BSI if HIGH-BIO agents are contraindicated.

**Disclosures:**

**All Authors**: No reported disclosures

